# An Assistive Coughing Device for Post-Laryngectomy Patients

**DOI:** 10.1109/TMRB.2021.3100798

**Published:** 2021-07-28

**Authors:** Keren Yue, Henry Lancashire, Kylie de Jager, James Graveston, Martin Birchall, Anne Vanhoestenberghe, Andrew Conn, Jonathan Rossiter

**Affiliations:** Bristol Robotics Laboratory and the Department of Engineering Mathematics, University of Bristol, United Kingdom; Department of Medical Physics and Biomedical Engineering, University College London, United Kingdom; Institute of Orthopaedics and Musculoskeletal Science, University College London, United Kingdom; The Ear Institute, University College London, United Kingdom; The Ear Institute, University College London, United Kingdom; Institute of Orthopaedics and Musculoskeletal Science, University College London, United Kingdom; Bristol Robotics Laboratory and the Department of Mechanical Engineering, University of Bristol, United Kingdom; Bristol Robotics Laboratory and the Department of Engineering Mathematics, University of Bristol, United Kingdom

## Abstract

People who have undergone total laryngectomy typically have difficulties speaking and coughing. Coughing, the protective reflex action where air is rapidly expelled from the lungs to clear the airway, is crucial in everyday life. Insufficiency in coughing can lead to serious chest infections. In this research we present a bionic assistive coughing device (RoboCough) to improve coughing efficacy among laryngectomy patients by increasing pressure and flow rate. RoboCough was designed to mimic the function of the glottis and trachea in the upper respiratory system. Experimental results show a significant increase (t(64) = 4.9, p < 0.0001) in peak cough flow rate and peak cough pressure (t(64) = 12.6, p < 0.0001) among 33 control participants using RoboCough. A pilot study with a smaller cohort of laryngectomy patients shows improvement in peak cough pressure (p = 0.0159) using RoboCough. Preliminary results also show that post-laryngectomy coughs achieved similar peak cough flow (Z = -0.9933, p = 0.32) to the control group’s natural cough. Coughing capabilities could be improved through using RoboCough. Applications of RoboCough include simulation of vocal folds and respiratory conditions, rehabilitation of ineffective coughs from laryngeal and respiratory diseases and as a test-bed for the development of medical devices for respiratory support.

## I Introduction

CANCER Research UK statistics reported around 11,900 new cases of head and neck cancers yearly between 2014-2016 [1]. Treatments of laryngeal cancer varies depending on the pathology. Total laryngectomy is indicated for the curative treatment of laryngeal cancer where the tumour is considered to be too advanced for radiotherapy or partial resection. Speech and coughing problems are significantly higher among patients with total laryngectomy compared to partial laryngectomy patients [2].

**Fig. 1:**
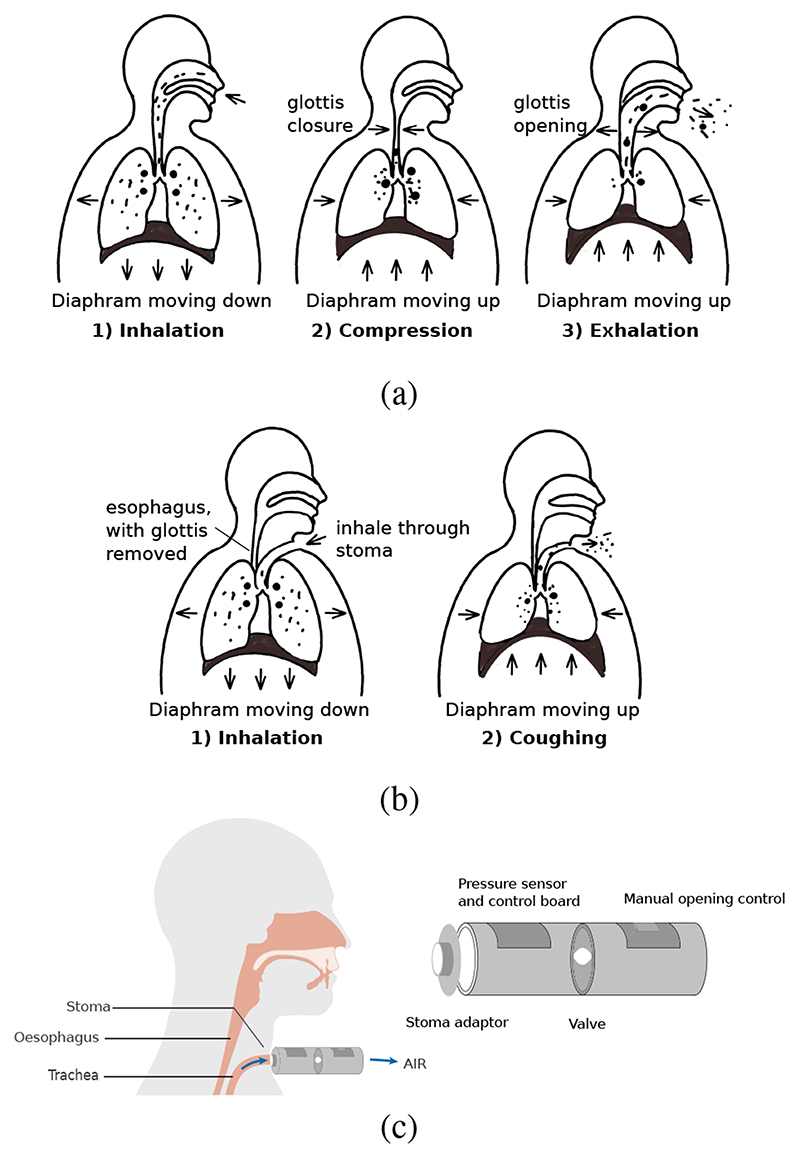
(a) Three phases of coughing in healthy people. (b) Coughing in laryngectomy patients is limited by the lack of a functional glottis. (c) Proposed cough assistive device.

**Fig. 2:**
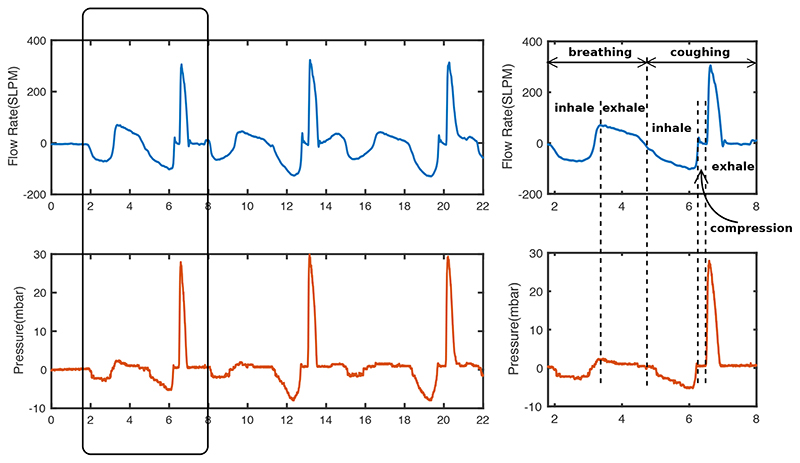
Flow rate and respiratory mouth pressure during three consecutive coughs by participants with a single cough profile enlarged on the right.

Total laryngectomy is a procedure that involves the removal of the entire larynx and the upper tracheal cartilage rings [3]. Post laryngecotomy patients are no longer at the risk of aspiration pneumonia, however some patients have difficulties in coughing. Coughing in healthy subjects, as can be seen from Fig 1a, typically involves three phases: inhalation, compression and exhalation. In inhalation phase, air is inhaled and expiratory muscles are engaged; In compression phase, glottis is closed, expiratory muscles contract, allowing intrathoracic pressure to build up in the airway; In exhalation phase, glottis opens and a rapid exhalation follows [4]. Expiratory pressure and flow profiles during coughs are shown in Fig 2. Cough is vital for clearing excessive secretions in the airway. However, the absence of the glottis in post-laryngectomy patients, and the direct exit of the trachea through the stoma, causes reduced cough efficiency (Fig 1b), such that daily sputum production, coughing and the need for frequent forced expectoration are among the most common complaints made by patients [5]. Although some patients find limited relief through the use of heat and moisture exchangers, which condition inspiratory air and thereby treating pulmonary symptoms and aid stoma cleaning [6], the coughing ability of post-laryngectomy patients is far below healthy people.

Clinically there are many potential factors that can be used to assess cough efficiency. For example, maximal respiratory mouth pressure and coughing gastric pressure are typically used to assess the strength of expiratory muscles [7], which are associated with coughing effectiveness [8]. The effectiveness of a cough can also be affected by the adhesion and cohesion of mucus [9], [10]. By simulating cough flow rates using a pressurized chamber, the influences of rheological properties on mucus clearance have been investigated [11]. Mechanical experiments have also shown that increasing the pressure and flow rate improves mucus clearance [12], and that increase in expiratory flow and pressure can be achieved using mechanical assistance for patients with motor-neuron diseases [13]. Studies show for effective mucus expectoration, peak cough flow (PCF) must exceed 160 to 200 L/min [14], [15]. PCF above 250 to 270 L/min could prevent pneumonia in patient with neuromuscular disorders [15], [16]. For laryngectomised patients, a lack of signals arising from the larynx may result in a reduction of cough volume acceleration and the intensity of abdominal muscle contractions during a reflex cough. These factors may contribute to facilitate the onset and/or the persistence of chest infections [17].

Medical robotics has advanced rapidly through past decades, from surgical robots such as the da Vinci surgical system and surgical tools developed for minimal invasive surgeries, to rehabilitation exoskeletons and implantable robotics such as heart sleeves [18] and drug delivery robots [19]. Despite this growth, there has been a dearth of research into robotics to assist respiratory conditions and, more specifically, cough insufficiency. Current cough augmentation methods generally fall into three categories: Mechanical insufflation-exsufflation (MI-E) technique such as Cough Assist, where a positive pressure is applied during inhalation and a fast negative pressure is applied for exhalation to improve cough, and rehabilitation devices to improve the cough flow [20]; Intermittent positive-pressure breathing (IPPB) devices which provide a constant positive pressure during the inhalation phase followed by a passive exhalation phase where the positive pressure returns to atmospheric, thereby improving cough capacity by increasing the inhalation volume [21]; and manually assisted coughing (MAC), where abdominal thrust or lateral costal compression is applied. Although these devices can effectively increase cough efficiency, they have a central limitation of poor portability and cannot be used during day-to-day activities. Additionally, most of the current devices are used in primary care settings and are operated by a care giver. Consequently, there is a large unmet need for cough assist devices in secondary and at-home care and there are currently no commercial devices available that offer independent user control and sufficient mobility for use during activities of daily living.

To overcome these limitations, we propose a highly portable user-operated device (Fig 1c) to improve cough sufficiency, which takes over glottal control while acting as an autonomous bionic “organ”. In this research a preliminary design of the RoboCough coughing device, that mimics the function of the glottis in the respiratory system, was built and tested.

## II Methods

Increased coughing efficiency is indicated by either an increased gastric pressure or an increased Peak Cough Flow rate (PCF), which can be achieved by increasing the expiratory muscle strength or by increasing the pressure resistance of the vocal folds. In this study we focus primarily on the function of the vocal fold area and hence expiratory pressure (peak cough pressure) and peak cough flow rate were measured and analysed. RoboCough is designed to have the following characteristics: fast opening and closing (< 30 ms [22]), airtight closure (able to withstand pressures up to 200 cmH_2_O), pressure-based feedback-controlled opening, fail safe opening mechanism and patient override.

### A Design of RoboCough

**Fig. 3:**
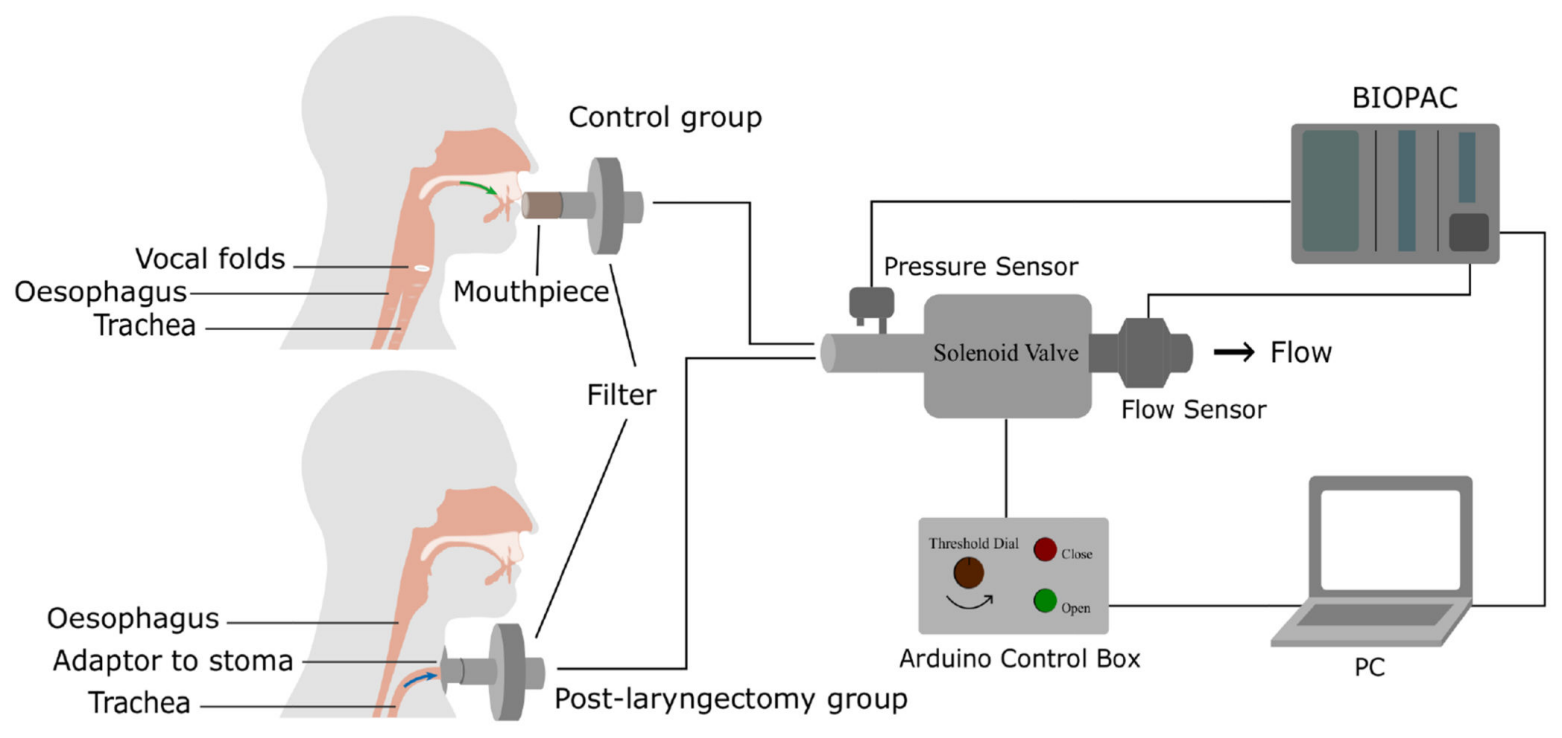
System design of RoboCough.

**Fig. 4:**
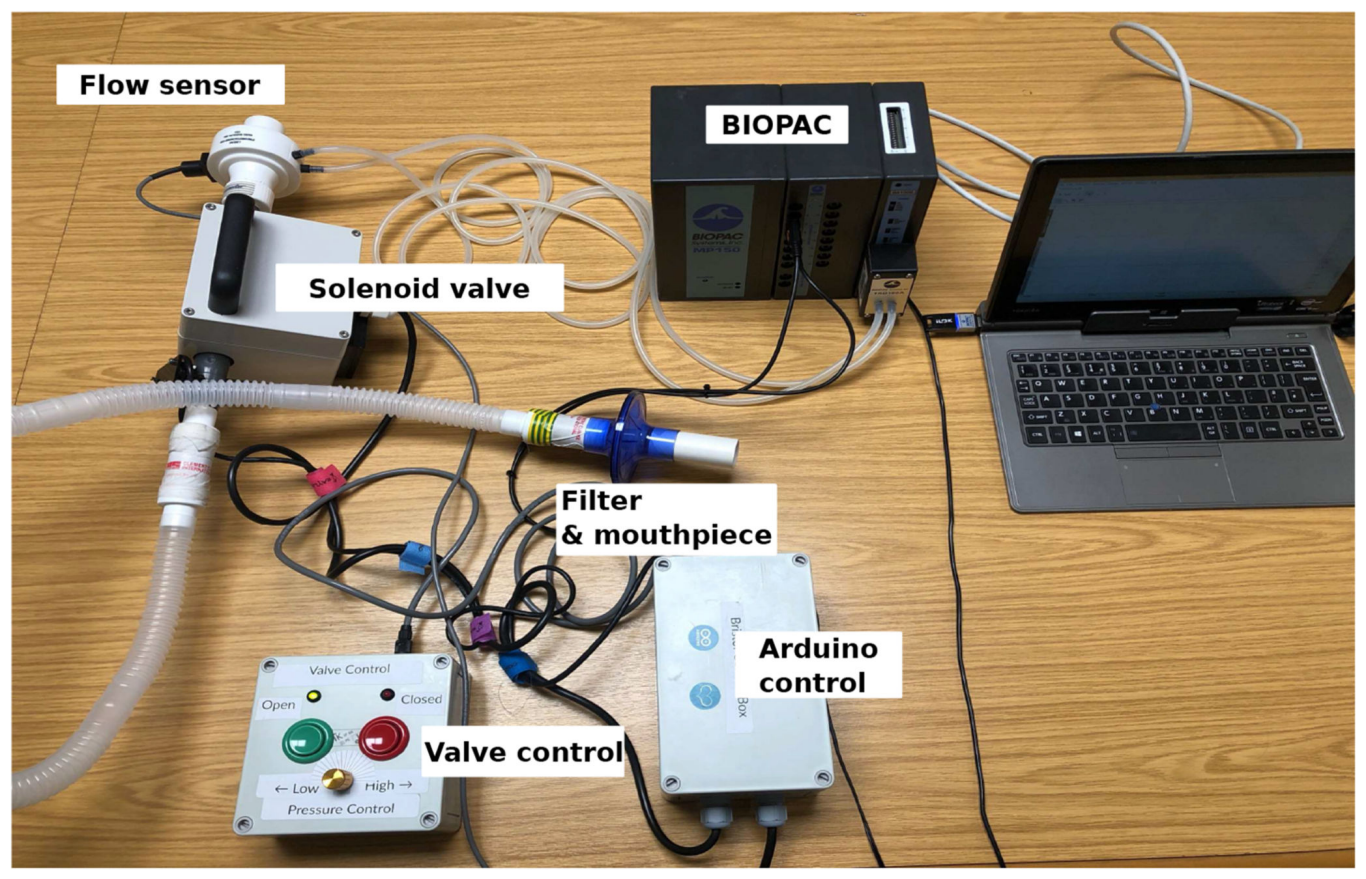
Photograph of the RoboCough test and evaluation system.

**Fig. 5:**
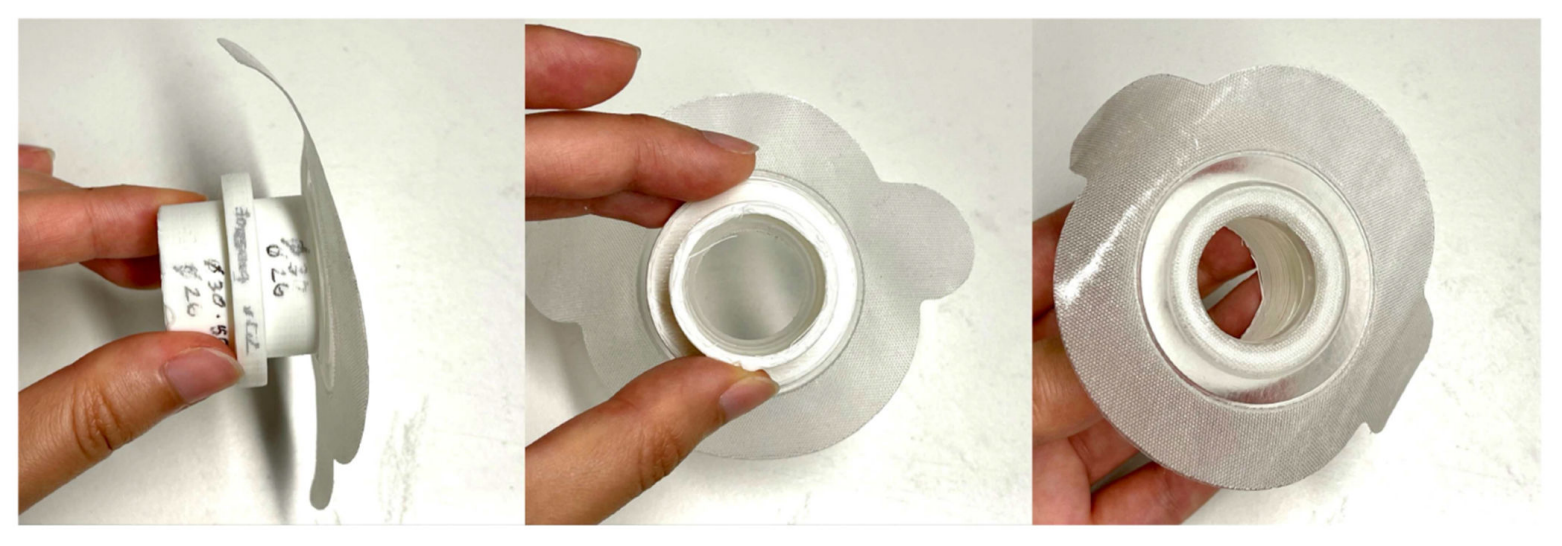
Photograph of the 3D printed stoma adaptor.

**Fig. 6:**
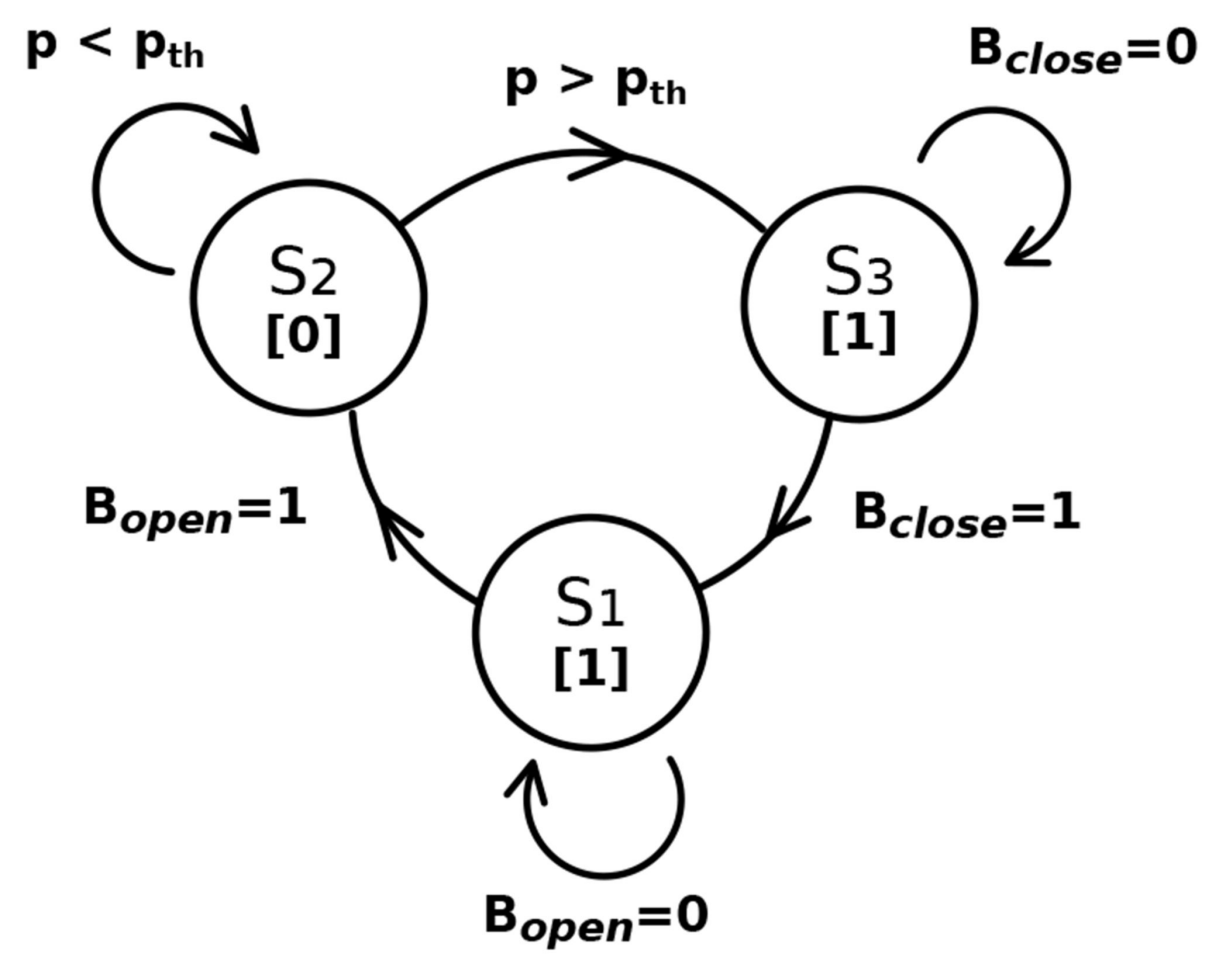
System state diagram relative to user input, where states *S_1_*,*S_2_* and *S_3_* correspond to the phases of inhale, compression and exhale (cough) shown in Fig 2, and valve states are represented by [0] for close and [1] for open. State transitions are governed by pressure threshold *p_th_* and button presses.

Fig 3a shows the RoboCough system design. Fig 4 shows a photograph of the device. A disposable cardboard mouthpiece was used to connect to an anti-bacterial and anti-viral filter (BIOPAC, AFT4 VBMax, 35mm), which was then connected to a normally open solenoid valve (ASCO SCE210C35, NO, 3/4in) via a respiratory tube (22mm diameter). For laryngectomy patient studies, a personalised 3D printed stoma adaptor was used in place of the cardboard piece to ensure an airtight connection around stoma. An example is shown in Fig 5, this stoma adaptor has a diameter of 26mm, and consists of a polylactic acid (PLA) component that push fit onto a standard medical-grade silicone stoma skin barrier. The solenoid valve was used to mimic the human glottis function during coughing. High speed photography analysis have shown the glottis opening time during is around 25-30 ms [22]. The chosen valve could achieve a comparable fast-opening time (15ms at fastest) which is also portable, low-cost and can maintain high pressure (9 bar), showing advantage over alternative valve types such as butterfly or ball valves, which are larger, slower or have lower maximum pressures. The solenoid valve’s orifice diameter is 19mm and its flow coefficient, *K_v_*, is 4.7m^3^/h. The estimated pressure loss Δ*p* caused by the valve can be calculated using the equation below, assuming air is incompressible [23]:

$$C_{v}=Q\sqrt{\frac{SG}{\text{Δ}p}},$$

where *Q* is the flow rate and *SG* is the specific gravity (0.0012 for air at 20°). For the selected valve, average Δ*p* is estimated to be 0.008 bar at average testing flowrate range. The pressure is monitored in real time by a pressure sensor (Honeywell SSCSNBN015PDAA5) in the respiratory tube before the valve, approximating peak cough pressure. The valve controls the air flow by sealing and building up pressure in the respiratory tube. During the inhalation phase, the valve opens, allowing air to be inhaled by participants. In the compression phase, the valve is activated and closes, allowing pressure to build up. In the exhalation phase, the valve opens again allowing air to be rapidly expelled. The valve automatically opens when pressure reaches a certain threshold (*p_th_*), mimicking the way vocal folds open during human cough. A Pneumotachometer (TSD137H) is attached to the expiratory flow end of the RoboCough to record cough flow rate using a BIOPAC MP150 data acquisition system. All tubing are of standard size (22mm) that used in clinical settings. Since pressure thresholds can vary from person to person and as coughing conditions vary, a dial located on the control box allows participants to alter the pressure threshold *p_th_* to a comfortable value. A manual override is available using two buttons on the control box (*B_close_* for closing and *B_open_* for opening the solenoid) so that users can immediately adjust the valve’s state at any time if they are not comfortable (this option was not observed during any of the tests in this study). The operation of the RoboCough, shown in Fig 6, is as follows: 1. The valve is open and the user inhales fully, (*S_1_*, valve [1]). 2. The valve is closed when the user presses the *B_close_* button (*S_2_*, valve [0]), replicating the closing of the glottis in preparation for a cough. 3. The user increases pressure in their lungs against the closed valve, generating the pressure needed for the cough. 4. When the pressure measured in the respiratory tube reaches *p_th_* the valve opens, rapidly releasing the pressurised air in the lungs and initiating an artificial cough, (*S_3_*, valve [1]). At any time, the user can press the *B_open_* button and RoboCough will immediate enter safe state *S_1_*.

### B Ethical Approvals

Ethical approval was obtained for this study from the University of Bristol Engineering Faculty Research Ethics Committee (ID:84502), and the University College London Research Ethics Committee (ID: 5697/006). Informed consent was sought from all participants before their involvement in the study following UoB and UCL guidance.

### C Subjects

33 control participants were recruited at University of Bristol and University College London (24 male and 9 female: ages 24 to 48, median age 28). Inclusion criteria for control participants were: any person between the age of 18 to 64, who does not have any condition or disease affecting their voice, and does not have any neuromuscular disorder. 5 post-laryngectomy participants were recruited at University College London (4 male and 1 female, ages 53 to 79, median age 72).

A power calculation was carried out using the software package “G*Power 3.1.9.2” [24] and data from an n = 5 control and m = 5 post-laryngectomy participant pilot study was used. Two tailed Wilcoxon signed-rank tests assumed, alpha = 0.05, beta = 0.8. A minimum sample size of 28 control participants was calculated for the difference in medians between RoboCough and voluntary coughs. A minimum sample size of 4 post-laryngectomy participants was calculated for the difference in peak cough pressure medians between RoboCough and voluntary cough, and 69 post-laryngectomy participants was calculated for the difference in peak cough flow medians between RoboCough and voluntary cough. Availability of post-laryngectomy participants restricted this group to m = 5.

### D Experiments

Experiments were carried out with n = 33 control participants and a pilot study was carried out with m = 5 post-laryngectomy participants to test the effectiveness of Robo-Cough. Peak Cough Flow (PCF, L/min), Peak Cough Pressure (PCP, cmH_2_O) and expiratory acceleration (L/*s^2^*) were measured during voluntary cough and RoboCough assisted cough. Results were compared between the control and the post-laryngectomy group. In both voluntary and RoboCough cough tests, the control group was provided with a mouthpiece whereas the post-laryngectomy group was provided with a 3D printed adapter which ensured a good seal around the stoma. Both groups were asked to breath in and out naturally through the mouthpiece/adapter several times to relax and to familiarise themselves with the device.

Participants were first asked to cough voluntarily with the valve kept open, giving a baseline for a peak voluntary cough. They were then asked to emulate a cough using RoboCough by performing a peak expiration manoeuvre. In both tests participants were asked to cough five consecutive times into the device. In tests using RoboCough, the cough pressure threshold was set by each participant to a level that they found comfortable.

In voluntary cough tests, the participant was instructed to cough as forcefully as they could (patient group users exhaled as forcefully as possible if unable to cough). In RoboCough cough tests, pressure threshold (*p_th_*) were fine tuned by each participant using the dial. Detailed instructions were given and participants were given time to tune the dial and attempt coughs at different pressure thresholds until they found a pressure threshold for which they could cough comfortably. For the control group, three different threshold settings were tested to ascertain its effect on cough effectiveness. Participants were asked to tune the threshold to mimic the situation of a “strong cough”, that is to cough using maximum effort. Then the threshold was tuned down to a medium level, to mimic the condition of a moderate cough. The threshold was then tuned down further to a point where participants felt it easy to cough, analogous to an instance of “throat clearing”. In the patient group, only one threshold setting was tested for safety reasons, when it felt most comfortable and natural to cough through the device.

### E Statistical analysis

All results are presented as mean ± standard deviation or median ± interquartile range where appropriate. Paired t-tests were used to determine differences in mean response to voluntary and RoboCough cough within the control group. Alpha cutoff was set to 0.05. Wilcoxon rank-sum tests were used to determine differences in mean response to voluntary and RoboCough cough within the post-laryngectomy group.

## III Results

**Fig. 7:**
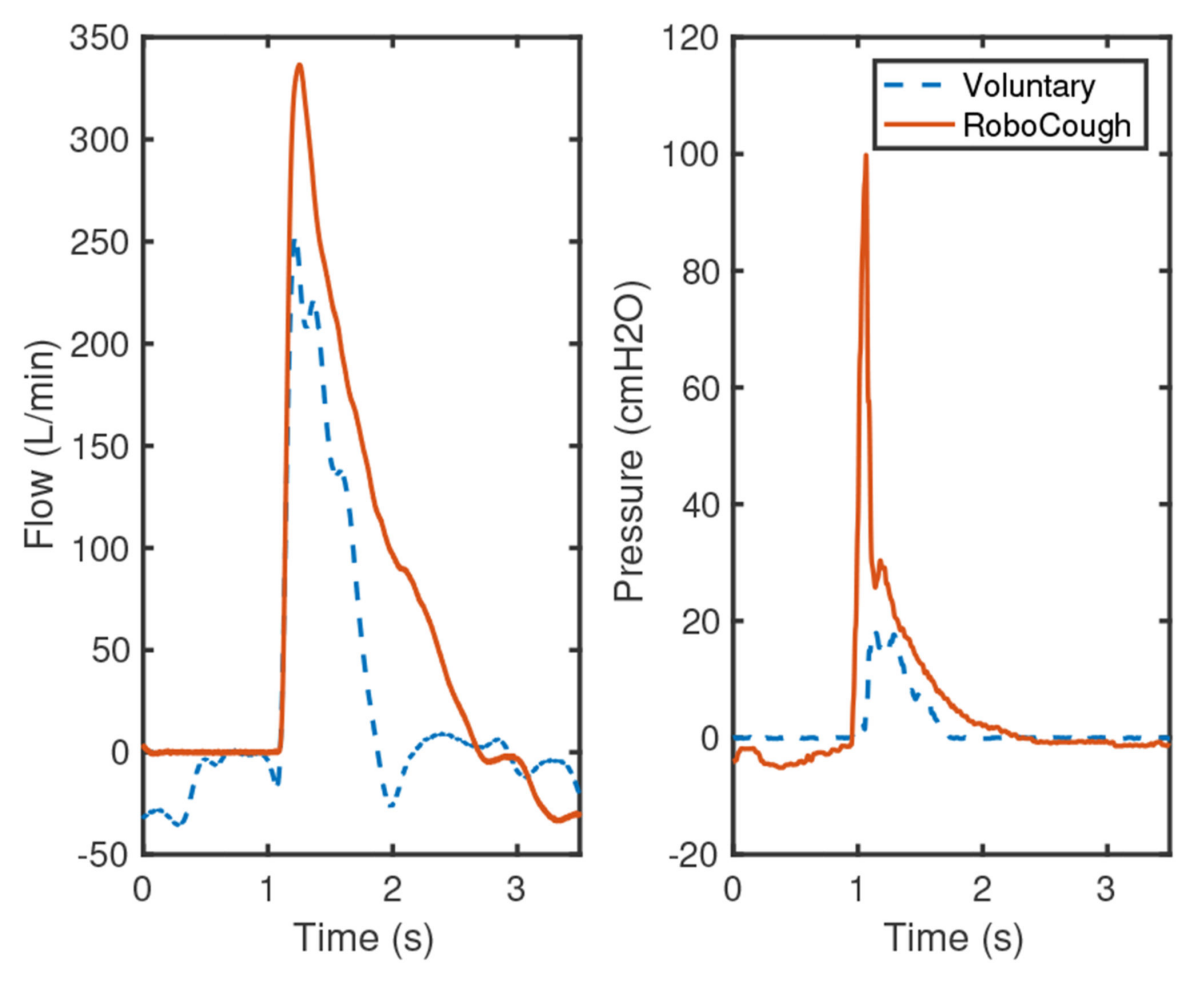
Example cough flow and pressure profiles from control group during voluntary and RoboCough assisted cough.

**Fig. 8:**
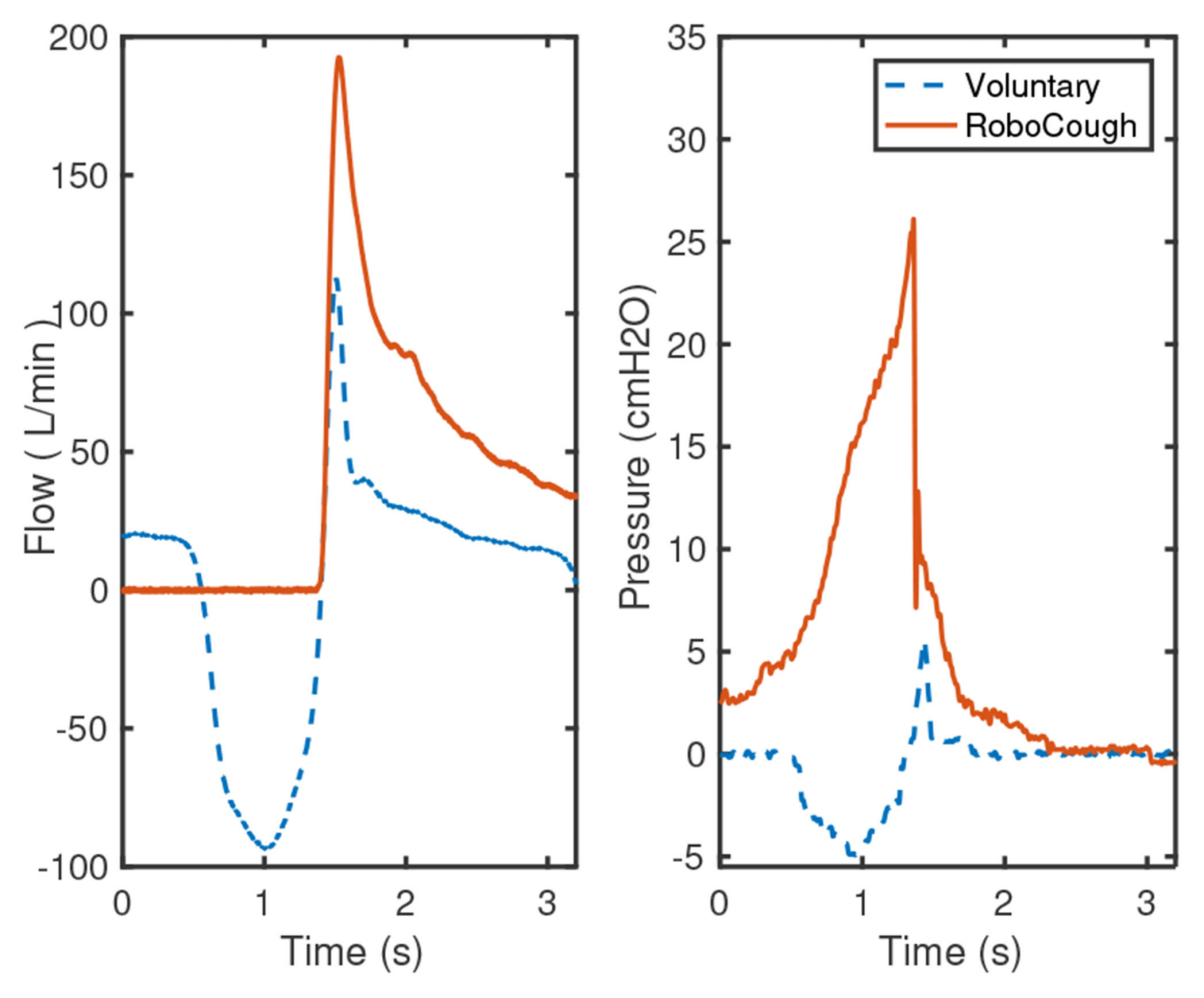
Example cough flow and pressure profiles from post-laryngectomy group during voluntary and RoboCough assisted cough.

Fig 7 and Fig 8 show a sample coughing profile of one participant from the control group and one from the post-laryngectomy group. It can be seen from the graphs that when comparing voluntary and RoboCough coughs, RoboCough generates a higher peak flow rate and a higher peak cough pressure, and takes a longer time to return to baseline. It is to be noted that the peak cough pressure measured in voluntary coughs is equivalent to MEP, while that recorded in RoboCough coughs is equivalent to glottal pressure. Cough acceleration, calculated as the ratio of cough peak flow to the time to peak, represented as the gradient rise to peak cough flow [25], [26], was also higher in RoboCough coughs.

In RoboCough experiments, only pressure and flow rate data were collected using the BIOPAC system. The pressure threshold was back-calculated from pressure and flow rate profiles based on the fact that the valve will only open when the pressure threshold is reached. It is assumed that the threshold pressure is reached when exhalation begins. To take into account delays in system processing and valve opening, a constant 80ms delay was assumed between reaching *p_th_* and the RoboCough actuating.

Table I summarizes the mean PCF, PCP and cough acceleration in all control group tests. As compared with voluntary coughs, all RoboCough tests generate higher mean PCF, PCP and acceleration. Within the RoboCough tests, the value of all parameters were seen to increase with pressure threshold.

**Fig. 9:**
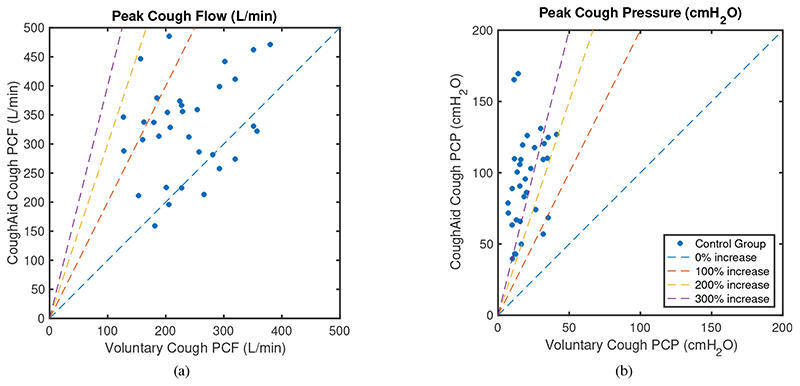
Mean peak cough flow (PCF) and peak cough pressure (PCP) with and without using RoboCough in control participants.

Fig 9 shows how PCF and PCP increase with the use of RoboCough in control group, with illustrative lines marking 0, 100, 200 and 300% increase. It can be seen that PCP was greater during RoboCough cough (94.4±32.7 cmH_2_O) than during voluntary cough (19.8±4.0 cmH_2_O) (paired t-test, t(64)=12.6, p<0.0001). PCF increased during RoboCough cough (329.1±82.9 L/min) compared with during voluntary cough (236.8±69.5 L/min) (paired t-test, t(64) = 4.9, p < 0.0001). Comparison between mean PCP and PCF with and without using RoboCough show that PCF increase can be found in 25/33 participants while PCP has increased significantly (p < 0.0001) in all participants. Peak cough flow differences before and during using RoboCough are further shown in Fig 11. Cough acceleration is also evaluated since it is one indicator on an effective cough. It has also increased significantly (t(64) = 4.39, p<0.01) using RoboCough, as shown in Table I.

**Fig. 10:**
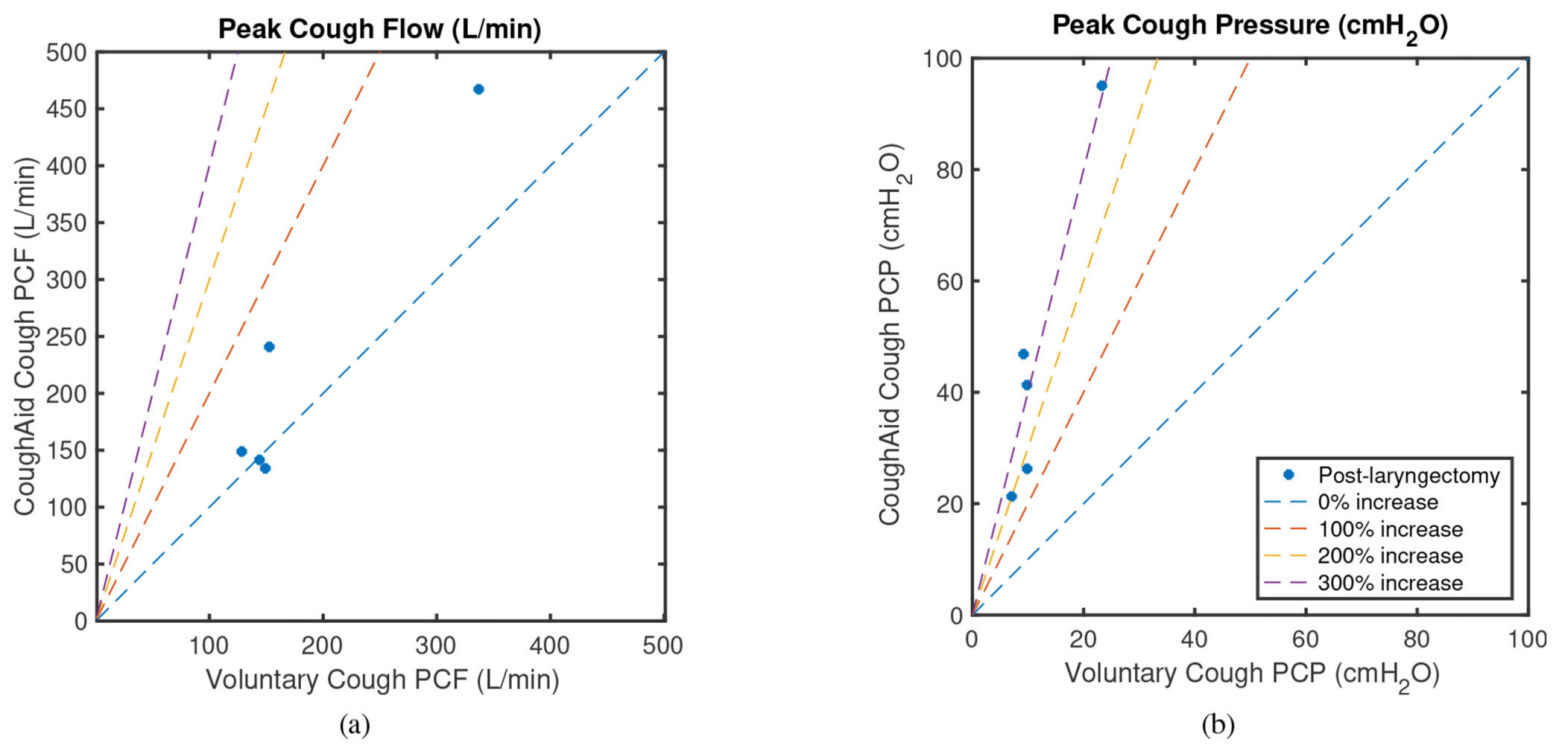
Mean peak cough flow (PCF) and peak cough pressure (PCP) with and without using RoboCough in post-laryngectomy participants.

**Fig. 11:**
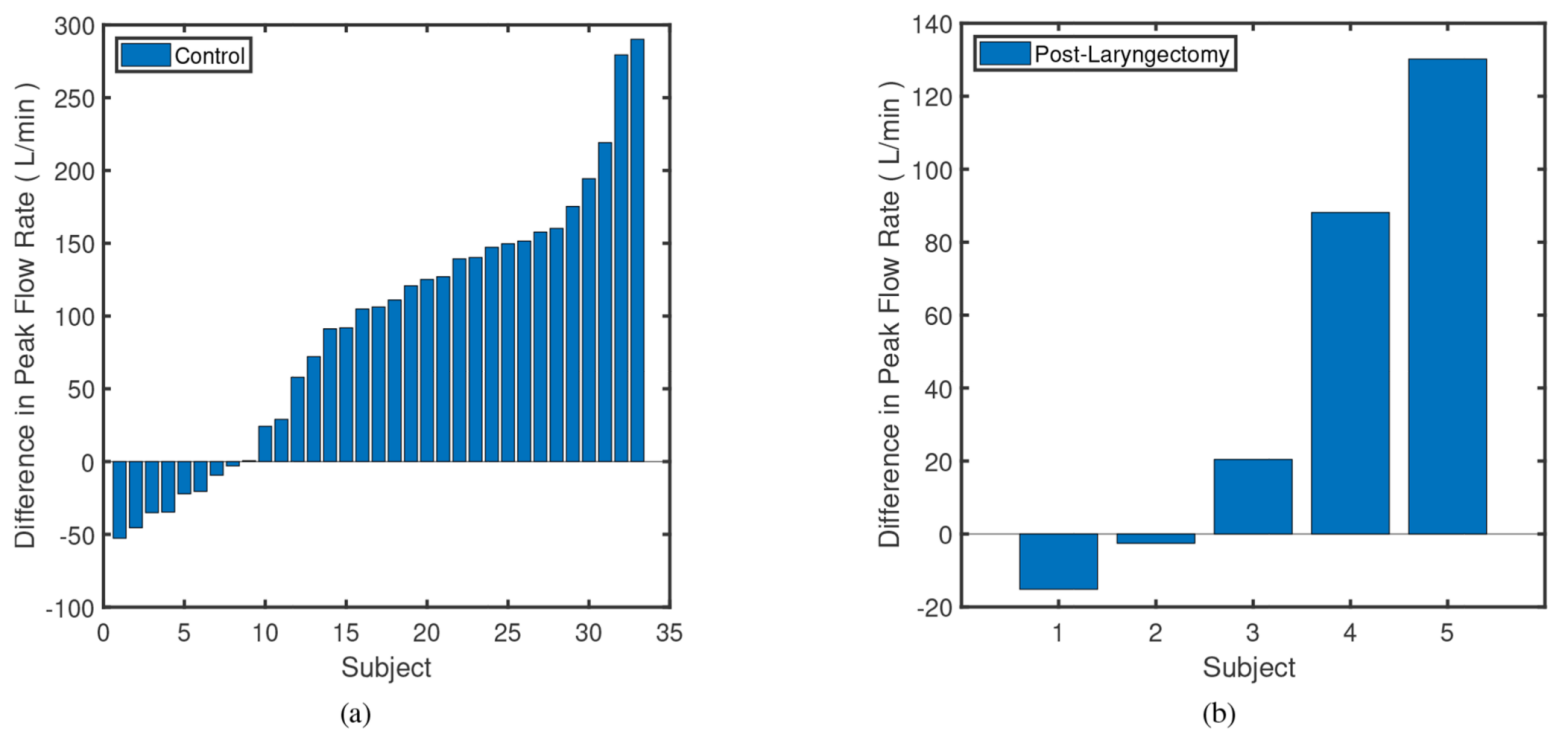
Difference in mean Peak Cough Flow in Control Group (Left) and Laryngectomy Group (Right), after using RoboCough.

Table II tabulates the mean PCP and PCF value in laryngectomy participants with and without RoboCough. Fig 10 shows how PCF and PCP increase with the use of RoboCough in the post-laryngectomy group, with illustrative lines marking 0, 100, 200, 300% increase. In these RoboCough tests, only one comfortable pressure threshold was set and tested for each post-laryngectomy participant. A Wilcoxon rank sum test shows a significant increase in pressure (p = 0.0159) with and without using RoboCough whereas peak flow (p = 1) does not show notable differences among the cohort. Results show an improvement in flow rate in 3/5 post-laryngectomy participants as can be seen more clearly from the ordered data in Fig 11.

When comparing data from post-laryngectomy group to control group, using the RoboCough, post-laryngectomy coughs also reached similar peak cough flow to the control group’s natural cough (Wilcoxon rank sum test, Z = -0.9933, p = 0.32), and achieved higher peak cough pressure (Wilcoxon rank sum test, Z = 2.6775, p = 0.0074).

## IV Discussion

In this study all cough data was analysed and there was no subjective data selection post experiments. The data collected therefore shows the natural dynamics of a series of voluntary and RoboCough coughs. We found that the RoboCough coughing assist device improved peak cough pressure and peak cough flow rate. The average percentage increase in peak flow rate was observed at 49% (paired t-test, t(64)=4.9, p<0.0001) in control group and 20% (Wilcoxon rank sum test, p = 1) in the post-laryngectomy group, while the average percentage increase in peak cough pressure was observed at 467% (paired t-test, t(64)=12.6, p<0.0001) in control group and 281% (Wilcoxon rank sum test, p = 0.0159) in the post-laryngectomy group. Note that the pressure sensor is located before the valve but after all tubing in the system. Measured pressure is therefore equivalent to expiratory pressure at the mouth in voluntary coughs and gastric pressure in RoboCough coughs. An effective cough can be dependent on several factors, including expiratory airflow rate, gasmucus interactions, mucus properties, the inhalation volume and expiratory muscle strengths [4], [27]. Studies show that inhaling high volumes, combined with glottis closure prior to the expiration phase, could enable respiratory muscles to generate high intrathoratic pressures. This pressure acts as a high driving force for the airflow during cough and hence contributes to high peak flow rate [27]. In the absence of the larynx (eg: laryngectomy, tracheotomy), coughing can still be generated with low airfow by performing a huffing manoeuvre (a forced expiration against open glottis), although the cough is less effective [4], [17], [28]. Maximum expiratory pressure is a commonly used parameter to assess the strength of respiratory muscles [7], [8], and has been shown to positively correlate with improved cough capacity [29]–[31].

In voluntary experiments, the peak cough pressure and flow from both control group and laryngectomy groups are lower than average published values in healthy adults [8],[17]. This is attributed to the tubing in the system as well as the presence of the solenoid valve restricting air flow. The 102cm respiratory tube represents a total dead volume in the RoboCough of approximately 0.39 litres, which is approximately 6.5 % of an average male’s lung capacity and 9.3 % of an average female’s lung capacity [32]. Other possible causes could be leakage around mouthpieces during coughing experiments and coughing while not fully prepared. Additionally, while undertaking the experiments, participants were asked to cough 5 times consecutively into the tube. This could result in a decrease in peak cough flow rate and pressure due to tiredness.

The control and post-laryngectomy groups had different age profiles, and age related factors were not taken into account when analysing the results. According to Cardoso et al [33], as age increases, peak cough flow decreases. However, the post-laryngectomy group (median age 72) had comparable PCP values to the control group (median age 28). This may be due to the post-laryngectomy group having stronger diaphram muscles than non-laryngecotomies of the same age group because they may use a technique called diaphragmatic breathing (also called abdominal breathing) to help breathing and coughing after laryngectomy [34].

In this study, pressure thresholds were set by each postlaryngectomy participant and were subjective and limited to voluntary cough manoeuvres. An alternative way of controlling the opening of the valve is to assess the activity of expiratory and accessory muscles through real-time EMG monitoring. Research have shown ways to characterise the difference between voluntary and reflex coughs using EMG signals [35]. The integration of an EMG signal into the valve control would expand the capability of the RoboCough to wider coughing scenarios. Replacing the solenoid valve with a valve with lower restriction could potentially increase cough effectiveness due to the reduced pressure drop across the valve [11]. Further investigation will also be carried out to quantify the relationship between peak cough pressure, threshold pressure, cough acceleration and peak flow rate produced by the RoboCough.

Results from this study show that RoboCough is a promising approach to increasing cough efficiency. It shows a comparable PCF increase to using MI-E and MI-E + MAC methods, although less effective than using IPPB + MAC in neuromuscular patients [14], [36]. RoboCough could be integrated in patient’s everyday life easily as it can be light weight, easy to use and each component are separable for handling. In rare cases where one has set the threshold pressure too high such that the valve may not open, the patient can either simply move the connector tube away to breathe, or press the emergency stop button and the valve will open. Scaling it down to the size of a peak flow meter (approximately 22cm long and 6cm wide) could effectively aid larygecotomy patients to cough when needed and the ultimate goal is to integrate RoboCough into a comfortable, wearable device.

## V Conclusion

This paper presents an approach to assist post laryngectomy patients cough more effectively. The rationale is that increasing the coughing pressure and flow rate would increase coughing effectiveness. This is usually achieved by increasing the strength of respiratory muscles, but this can take significant time and training effort, and without a natural glottis, coughing effectiveness remains low. In this study, we explored an novel alternative, where an increase in the strength of an artificial glottis closure increases coughing pressure and peak air flow. To achieve this we designed and built the RoboCough, which mimics the function of the glottis. We then tested and compared the peak cough flow and peak cough pressure in control and post-laryngectomy groups through a series of respiratory experiments. Results show a significant increase in peak cough pressure, flow rate and acceleration in the control group. Using the RoboCough, post-laryngectomy coughs achieved similar peak cough flow to the control group’s natural cough, showing an improvement in their coughing capabilities. As a first step towards a fully autonomous and bio-integrated bionic device for full function restoration, this work paves the way for new treatments for cough insufficiency in respiratory disease sufferers and may additionally have potential for the rehabilitation of coughing following respiratory conditions such as COVID19.

## Figures and Tables

**Table I:** Summary of coughing characteristics in control group. (Bold shows statistically significant data.)

	Peak Cough Flow (L/min)	Peak Cough Pressure (cmH_2_O)	Acceleration (L/*s^2^*)
Voluntary Cough	236.8 ± 69.5	19.8 ± 4.0	45.3 ± 13.5
Low *p_th_* RoboCough	279.3 ± 67.1	58.8 ± 18.2	74.2 ± 14.1
Medium *p_th_* RoboCough	290.6 ± 74.3	69.1± 22.1	78.5 ± 14.5
High *p_th_* RoboCough	**329.1** ± **82.9**	**94.4** ± **32.7**	**91.0** ± **19.0**

**Table II:** Coughing Characteristics in Post-Laryngectomy Group. (Bold Shows Statistically Significant Data.)

	Peak Cough Flow (L/min)	Peak Cough Pressure (cmH_2_O)	Acceleration (L/*s^2^*)
Voluntary Cough	182.3 ± 86.9	11.9 ± 6.5	36.4 ± 14.6
RoboCough	226.5 ± 141.4	**46.2** ± **29.3**	**115.9** ± **165.9**
